# Association of Antepartum and Postpartum Air Pollution Exposure With Postpartum Depression in Southern California

**DOI:** 10.1001/jamanetworkopen.2023.38315

**Published:** 2023-10-18

**Authors:** Yi Sun, Kathryne S. Headon, Anqi Jiao, Jeff M. Slezak, Chantal C. Avila, Vicki Y. Chiu, David A. Sacks, John Molitor, Tarik Benmarhnia, Jiu-Chiuan Chen, Darios Getahun, Jun Wu

**Affiliations:** 1 Institute of Medical Information, Chinese Academy of Medical Sciences and Peking Union Medical College, Beijing, China; 2 Department of Environmental and Occupational Health, Program in Public Health, University of California, Irvine; 3 School of Medicine, University of California, Irvine; 4 Department of Research and Evaluation, Kaiser Permanente Southern California, Pasadena, California; 5 Department of Obstetrics and Gynecology, Keck School of Medicine, University of Southern California, Los Angeles, California; 6 College of Public Health and Human Sciences, Oregon State University, Corvallis, Oregon; 7 Scripps Institution of Oceanography, University of California, San Diego; 8 Departments of Population and Public Health Sciences and Neurology, Keck School of Medicine, University of Southern California, Los Angeles, California; 9 Department of Health Systems Science, Kaiser Permanente Bernard J. Tyson School of Medicine, Pasadena, California

## Abstract

**Importance:**

Women are especially vulnerable to mental health matters post partum because of biological, emotional, and social changes during this period. However, epidemiologic evidence of an association between air pollution exposure and postpartum depression (PPD) is limited.

**Objective:**

To examine the associations between antepartum and postpartum maternal air pollution exposure and PPD.

**Design, Setting, and Participants:**

This retrospective cohort study used data from Kaiser Permanente Southern California (KPSC) electronic health records and included women who had singleton live births at KPSC facilities between January 1, 2008, and December 31, 2016. Data were analyzed between January 1 and May 10, 2023.

**Exposures:**

Ambient air pollution exposures were assessed based on maternal residential addresses using monthly averages of particulate matter less than or equal to 2.5 μm (PM_2.5_), particulate matter less than or equal to 10 μm (PM_10_), nitrogen dioxide (NO_2_), and ozone (O_3_) from spatial interpolation of monitoring station measurements. Constituents of PM_2.5_ (sulfate, nitrate, ammonium, organic matter, and black carbon) were obtained from fine-resolution geoscience-derived models based on satellite, ground-based monitor, and chemical transport modeling data.

**Main Outcomes and Measures:**

Participants with an Edinburgh Postnatal Depression Scale score of 10 or higher during the 6 months after giving birth were referred to a clinical interview for further assessment and diagnosis. Ascertainment of PPD was defined using a combination of diagnostic codes and prescription medications.

**Results:**

The study included 340 679 participants (mean [SD] age, 30.05 [5.81] years), with 25 674 having PPD (7.54%). Increased risks for PPD were observed to be associated with per-IQR increases in antepartum and postpartum exposures to O_3_ (adjusted odds ratio [AOR], 1.09; 95% CI, 1.06-1.12), PM_10_ (AOR, 1.02; 95% CI, 1.00-1.04), and PM_2.5_ (AOR, 1.02; 95% CI, 1. 00-1.03) but not with NO_2_; PPD risks were mainly associated with PM_2.5_ organic matter and black carbon. Overall, a higher risk of PPD was associated with O_3_ during the entire pregnancy and postpartum periods and with PM exposure during the late pregnancy and postpartum periods.

**Conclusions and Relevance:**

The study findings suggest that long-term exposure to antepartum and postpartum air pollution was associated with higher PPD risks. Identifying the modifiable environmental risk factors and developing interventions are important public health issues to improve maternal mental health and alleviate the disease burden of PPD.

## Key Points

**Question:**

Is maternal ambient air pollution exposure associated with increased risks of postpartum depression (PPD)?

**Findings:**

In this cohort study of 340 679 pregnant women, antepartum and postpartum exposures to ozone, particulate matter less than or equal to 10 μm, and particulate matter less than or equal to 2.5 μm and its constituents (organic matter and black carbon) were associated with increased risks of PPD.

**Meaning:**

These findings suggest that long-term antepartum and postpartum air pollution exposure is a potentially modifiable environmental risk factor for PPD and an important public health issue to address for improved maternal mental health.

## Teaser

This cohort study examines the associations between antepartum and postpartum air pollution exposure and postpartum depression in mothers living in Southern California.

## Introduction

Postpartum depression (PPD) is the main perinatal form of major depressive disorder and is one of the most frequent childbirth complications.^1^ Postpartum depressive symptoms affect approximately 10% to 20% of women worldwide,^1,2^ and approximately 9% of mothers experience PPD based on validated diagnostic interviews.^3^ Mothers with PPD experience symptoms such as depressed mood, anxiety, and anhedonia^4^; have a higher risk of morbidity and mortality from suicide; and may be more likely to commit infanticide.^5,6^ Infants born to mothers with PPD may be at a higher risk of developing cognitive, emotional, and psychological impairments and behavioral abnormalities.^5,7^ Identifying modifiable environmental risk factors is important due to increased susceptibility during the antepartum and postpartum periods.

Previous research has shown that air pollution exposure may contribute to several health concerns, including neuropsychological disorders,^8,9,10^ and could result in subsequent poor mental health. Air pollution’s impact on mental health has been hypothesized to be due to several biological mechanisms, including the hypothalamic-pituitary-adrenal (HPA) axis dysfunction, chronic inflammation, and oxidative stress–mediated neuropathology.^7,8,10,11,12,13^

Growing epidemiologic evidence has shown a link between air pollution and mental health disorders among the general population.^14,15,16^ However, few studies have examined the association between air pollution exposure and PPD, and these studies have reported inconsistent findings.^4,17,18,19^ Inconclusive results may be partially due to differences in study populations, sample sizes, methods of outcome assessments, exposure windows, and study regions with different levels and compositions of particulate matter less than or equal to 2.5 μm (PM_2.5_). For example, 2 studies performed in the US had very small samples (n = 598 and 180) and, thus, may lack statistical power.^17,18^ Maternal depression or depressive symptoms in previous studies were defined using questionnaires, such as the Edinburgh Postnatal Depression Scale (EPDS) and Center for Epidemiologic Studies-Depression Scale, without further assessment and clinical diagnosis of PPD. No previous study has examined PM_2.5_ chemical compositions, which have large spatiotemporal variations and affect health differently.^20^ Furthermore, air pollution exposures in prior studies focused on the antepartum period or were estimated based only on one-time surveys (eg, 6 weeks, 6 months, or 12 months post partum) without considering different durations of time-varying exposures, potentially leading to bias in the exposure estimates. Pregnancy is a sensitive period, as women experience major metabolic changes, and pregnancy-induced hyperventilation may lead to inhalation of more ambient toxic substances.^21^ Considering susceptible windows of exposure could be as important as considering the intensity of exposure. Investigating the critical exposure time windows and different air pollution constituents could help to reveal etiologic mechanisms and aid in the design of targeted interventions or guidance on behavioral changes for high-risk women during and after pregnancy.^22^ In this study, we aimed to (1) investigate the associations between PPD and maternal residential exposure to air pollution (ie, PM_2.5_, particulate matter less than or equal to 10 μm [PM_10_], nitrogen dioxide [NO_2_], ozone [O_3_], and PM_2.5_ constituents) in a large pregnancy cohort in Southern California and (2) identify windows of susceptibility to antepartum and postpartum air pollution exposure.

## Methods

### Study Population

This retrospective cohort study was approved by the Kaiser Permanente Southern California (KPSC) institutional review board with an exemption of informed consent, as the research was considered minimal risk for participants. This study followed the Strengthening the Reporting of Observational Studies in Epidemiology (STROBE) reporting guideline. Data were analyzed between January 1 and May 10, 2023.

This cohort included women who had births between January 1, 2008, and December 31, 2016, at KPSC facilities. The KPSC serves approximately 19% of the population in Southern California and represents its sociodemographic diversity, which provides valid inference for epidemiologic research.^23,24^ Women who were not a KPSC member, who gave birth at less than 20 weeks or more than 47 weeks gestation, who did not have a residential address, who’d had multiple births, and who had deliveries resulting in a stillbirth were excluded from the study. The KPSC electronic health records (EHRs) provided detailed information on demographic characteristics, medical records, birth records, and individual lifestyles.

### Outcome

Postpartum depression was first assessed using the EPDS for participants during their postpartum visits.^25^ Participants with positive screening results based on the EPDS (score ≥10) during postpartum visits were referred to a clinical interview for further assessment and follow-up care, including diagnosis and treatment.^26^ Compared with the PPD diagnosis solely based on questionnaires or diagnostic codes in EHRs, the completeness and accuracy of PPD identification can be improved by supplementing clinical codes with pharmacy use records.^27^ Therefore, PPD diagnosis in this study was defined by using a combination of *International Classification of Diseases, Ninth* and *10th Revision* diagnostic codes for depression and prescription medication records (eTable 1 in Supplement 1) from the date of delivery to the first 6 months after delivery in KPSC EHRs.

### Exposure Assessment

Daily ambient air pollution measurements for NO_2_, O_3_ (an 8-hour window of 10 am-6 pm), PM_2.5_, and PM_10_ from 2007 to 2017 were obtained from the US Environmental Protection Agency’s monitoring stations. Monthly averages were then calculated and spatially interpolated between stations using empirical Bayesian kriging (EBK).^28,29^ Publicly available monthly data (1 × 1-km resolution) on PM_2.5_ total mass and constituents, including sulfate, nitrate, ammonium, organic matter, and black carbon, were derived from the fine-resolution geoscience-derived model, which combined satellite remote sensing data, ground-based measurements, and chemical transport modeling to provide validated PM_2.5_ outputs over North America.^30,31^ More details are described in the eMethods in Supplement 1 and elsewhere.^32^

Information on residential changes during pregnancy (address, start date, and end date) was abstracted from KPSC EHRs. All maternal residential addresses were geocoded. Monthly air pollution estimates during pregnancy were spatiotemporally linked to each woman based on the geocoded residential history; air pollution exposures during postpartum periods were estimated based on the residential address at delivery. We then temporally interpolated the monthly air pollution metrics to generate daily values and calculated trimester-specific and postpartum exposures by averaging the air pollution measurements in each specific period (more details are provided in the eMethods in Supplement 1). Long-term antepartum and postpartum air pollution exposure was defined as the period from conception to the date of PPD diagnosis within 6 months after delivery.

### Covariates

Pregnancy-related covariates and potential confounders were selected a priori based on the existing literature^2,4,17,18,19^ were obtained from KPSC EHRs, and included maternal age, self-reported race and ethnicity, and educational level; smoking status during pregnancy; exposure to passive smoking; season of conception; and year of infant birth (2008-2016). Block group–level median household income was estimated using the 2013 census tract based on patients’ residential address at delivery (categorized as quartiles).^33^

### Statistical Analysis

The distribution of selected population characteristics and air pollution metrics were assessed. We used χ^2^ tests to determine the difference between PPD and non-PPD groups. Pearson correlation was applied to examine the correlation between air pollution metrics. The association between PPD and air pollution was examined using time-to-event models, and women without PPD were censored at 6 months after delivery. Specifically, we used a discrete-time approach with pooled logistic regressions to estimate time-varying associations between air pollution exposure and PPD during each period, including the entire pregnancy, trimesters, and postpartum periods. A discrete-time approach is more flexible than traditional approaches that require the proportional hazards assumption (eg, Cox proportional hazards regression) and may be useful for handling large data sets with time-dependent variables. In discrete-time logistic regression models,^34^ we included time (the gestational month) as a covariate in a flexible manner (polynomials), as suggested by prior research.^16^ In trimester-specific analysis, we included all trimester average exposures in a single model to reduce bias as suggested in a previous study.^35^ Odds ratios (ORs) and 95% CIs of PPD associated with per-IQR increment of each air pollutant were estimated.

The primary analysis was adjusted for maternal age, race and ethnicity, education, median neighborhood household income, smoking during pregnancy, season of conception, and year of infant birth. County was fitted as a random effect to account for potential PPD spatial clustering. In sensitivity analyses, we examined the influence of further controlling for pregnancy-related comorbidities and preterm birth, preconception air pollution levels, green space exposure, multiple deliveries, fixed cohort bias, and urban and rural status (more details are provided in the eMethods in Supplement 1).

Due to potential differences in susceptibility of air pollution effects on health across population subgroups,^36^ we performed stratified analyses to examine whether the hypothesized associations differed by maternal characteristics, including maternal age, race and ethnicity, educational level, neighborhood household income, and prepregnancy body mass index categories. Cochran *Q* tests were used to measure the heterogeneity among subgroups. Adjustment for multiple comparisons was not made for subgroup or sensitivity analyses; thus, those results should be interpreted as exploratory. Participants with missing or invalid data for the exposure (eg, living in regions where air pollution could not be calculated) or confounding variables were excluded from the analysis. All analyses were conducted using SAS, version 9.4 statistical software (SAS Institute Inc). A 2-sided *P* < .05 was considered statistically significant.

## Results

A total of 340 679 singleton births (eFigure in Supplement 1), including 25 674 mothers with PPD (7.54%), at 6 months after delivery were included in our study. Among women with PPD, 9238 (35.98%) had both a PPD diagnosis and prescription medications, 6290 (24.50%) were identified by diagnostic codes solely, and 10 146 (39.52%) were identified by supplemental pharmacy records. The mean (SD) maternal age of the study population was 30.05 (5.81) years at delivery. Hispanic mothers accounted for the majority (50.86%) of the total population (vs 7.76% African American, 12.46% Asian, 26.39% non-Hispanic White, and 2.53% multiple or other races and ethnicities [including American Indian or Alaska Native and Pacific Islander]). Table 1 presents the distribution of population characteristics by PPD groups. Postpartum depression vs no PPD was more frequent among older (mean [SD] age, 30.97 [5.62] vs 29.98 [5.82] years), African American (8.18% vs 7.73%), and non-Hispanic White (34.70% vs 25.71%) mothers; mothers with less than 4 years of college (26.17% vs 22.36%) or more than a college education (13.97% vs 12.46%); mothers living in high-income neighborhoods (27.13% vs 24.73%) and rural areas (3.80% vs 3.40%); mothers with overweight (28.11% vs 27.62%) or obesity (32.18% vs 24.94%); and mothers who smoked during pregnancy (8.96% vs 5.19%) (all *P* < .001).

**Table 1.** Selected Population Characteristics by Postpartum Depression (PPD) Status, 2008-2016

Characteristic	No. (%)			
	PPD (n = 25 674)	Non-PPD (n = 315 005)	Total births (N = 340 679)
Maternal age, mean (SD), y	30.97 (5.62)	29.98 (5.82)	30.05 (5.81)
Maternal race and ethnicity			
African American	2100 (8.18)	24 338 (7.73)	26 438 (7.76)
Asian	1558 (6.07)	40 879 (12.98)	42 437 (12.46)
Hispanic	12 375 (48.20)	160 887 (51.07)	173 262 (50.86)
Non-Hispanic White	8908 (34.70)	80 996 (25.71)	89 902 (26.39)
Multiple or other^a^	732 (2.85)	7872 (2.50)	8604 (2.53)
Missing	1 (0.01)	35 (0.01)	36 (0.01)
Maternal education			
Less than college	7314 (28.49)	103 073 (32.72)	110 387 (32.40)
College (<4 y)	6720 (26.17)	70 423 (22.36)	77 143 (22.64)
College (≥4 y)	7599 (29.60)	96 575 (30.66)	104 174 (30.58)
More than college	3587 (13.97)	39 234 (12.46)	42 821 (12.57)
Missing	454 (1.77)	5700 (1.81)	6154 (1.81)
Median household income at block group level			
<$43 644	5439 (21.18)	79 505 (25.24)	84 944 (24.93)
$43 644-$55 833	6337 (24.68)	78 467 (24.91)	84 804 (24.89)
$55 834-$71 429	6837 (26.63)	77 975 (24.75)	84 812 (24.89)
>$71 429	6966 (27.13)	77 894 (24.73)	84 860 (24.91)
Missing	95 (0.37)	1164 (0.37)	1259 (0.37)
Smoking during pregnancy			
Never	19 062 (74.25)	263 836 (83.76)	282 898 (83.04)
Ever	4310 (16.79)	34 788 (11.04)	39 098 (11.48)
Smoked during pregnancy	2301 (8.96)	16 339 (5.19)	18 640 (5.47)
Missing	1 (0.01)	42 (0.01)	43 (0.01)
Passive smoker			
Yes	705 (2.75)	7115 (2.26)	7820 (2.30)
No	24 901 (96.99)	305 943 (97.12)	330 844 (97.11)
Missing	68 (0.26)	1947 (0.62)	2015 (0.59)
Prepregnancy BMI in categories			
Underweight (<18.5)	440 (1.71)	8211 (2.61)	8651 (2.54)
Normal (18.5-24.9)	9601 (37.40)	138 083 (43.84)	147 684 (43.35)
Overweight (25.0-29.9)	7216 (28.11)	86 996 (27.62)	94 212 (27.65)
Obesity (≥30.0)	8263 (32.18)	78 563 (24.94)	86 826 (25.49)
Missing	154 (0.60)	3152 (1.00)	3306 (0.97)
Season of conception			
Warm season (May-October)	12 698 (49.46)	155 559 (49.38)	168 257 (49.39)
Cool season (November-April)	12 976 (50.54)	159 446 (50.62)	172 422 (50.61)
Rural and urban status			
Rural	976 (3.80)	10 707 (3.40)	11 683 (3.43)
Urban	24 698 (96.20)	304 298 (96.60)	328 996 (96.57)
Year of infant birth			
2008	2123 (8.27)	33 263 (10.56)	35 386 (10.39)
2009	2150 (8.37)	31 740 (10.08)	33 890 (9.95)
2010	2172 (8.46)	31 589 (10.03)	33 761 (9.91)
2011	2335 (9.09)	33 260 (10.56)	35 595 (10.45)
2012	2440 (9.50)	35 614 (11.31)	38 054 (11.17)
2013	3143 (12.24)	35 470 (11.26)	38 613 (11.33)
2014	3774 (14.70)	36 292 (11.52)	40 066 (11.76)
2015	3845 (14.98)	38 024 (12.07)	41 869 (12.29)
2016	3692 (14.38)	39 753 (12.62)	43 445 (12.75)

Abbreviation: BMI, body mass index (calculated as weight in kilograms divided by height in meters squared).

^a^ Includes Pacific Islander, American Indian or Alaska Native, and mothers with multiple races and ethnicities specified by the patient.

### Correlations Among Air Pollutants

Summary statistics and Pearson correlation coefficients between air pollution metrics are shown in eTable 2 and the eResults in Supplement 1. Kriged PM_2.5_ was positively and moderately correlated with PM_10_ (*r* = 0.66) and NO_2_ (*r* = 0.64), while O_3_ was negatively correlated with PM_2.5_ (*r* = −0.11) and NO_2_ (*r* = −0.33) and weakly correlated with PM_10_ (*r* = 0.23). The PM_2.5_ mass concentrations from the EBK model and the chemical constituent model were highly correlated (*r* = 0.82), and both were moderately or highly correlated with PM_2.5_ constituents (*r* = 0.58-0.93).

### Associations Between Air Pollution and PPD and Critical Exposure Windows

The associations between antepartum and postpartum exposure to air pollution and the risk of PPD are shown in the Figure. Positive associations were observed between PPD and kriged PM_2.5_, PM_10_, and O_3_. The adjusted OR per IQR increase was strongest for O_3_ (1.09; 95% CI, 1.06-1.12), followed by PM_10_ (1.02; 95% CI, 1.00-1.04) and PM_2.5_ (1.02; 95% CI, 1. 00-1.03). No statistically significant association was observed between NO_2_ and PPD. For PM_2.5_ chemical constituent models, exposure to PM_2.5_ total mass, organic matter, and black carbon were associated with increased PPD risks. The strongest association was observed for PM_2.5_ black carbon (OR, 1.04; 95% CI. 1.00-1.09). In sensitivity analyses (eTable 3 in Supplement 1), the results were similar to the primary analysis after controlling for green space, preconception air pollution, or the random effect of multiple deliveries. Associations of air pollution with PPD were slightly decreased in magnitude after further adjusting for pregnancy-related comorbidities and preterm birth, while slightly stronger after restricting to urban areas or controlling for fixed cohort bias compared with the primary analysis.

**Figure.**
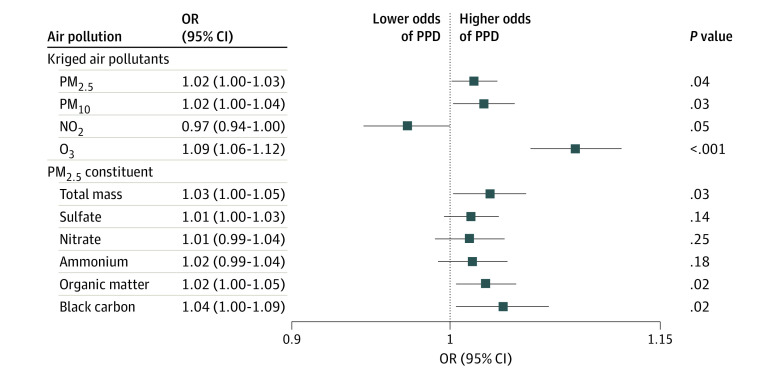
Associations Between Antepartum and Postpartum Air Pollution Exposure and Postpartum Depression (PPD) Odds ratios (ORs) and 95% CIs were calculated per IQR increment for each air pollutant. The model was adjusted for maternal age, race and ethnicity, education, median neighborhood household income, smoking during pregnancy, season of conception, and year of infant birth. County was fitted as a random effect. NO_2_ indicates nitrogen dioxide; O_3_, ozone; PM_2.5_, particulate matter less than of equal to 2.5 μm; PM_10_, particulate matter less than or equal to 10 μm.

In terms of antepartum air pollution exposure (Table 2), we only found kriged O_3_ during the entire pregnancy to be associated with PPD (adjusted OR, 1.07; 95% CI, 1.04-1.10). When considering PM_2.5_ constituents, we found an increased PPD risk with PM_2.5_ organic matter and black carbon during the entire pregnancy but no associations with other PM_2.5_ constituents. The trimester-specific results showed that increased risk was detected in the first and third trimesters for kriged O_3_ and in the third trimester for PM_10_ and PM_2.5_. Associations of O_3_ and PM exposure with PPD were slightly stronger after combining air pollution exposure in the third trimester and the postpartum period. Moreover, associations between different PM_2.5_ constituents and PPD differed by exposure windows (Table 2). Increased risks of PPD were observed for PM_2.5_ black carbon during the first trimester and PM_2.5_ nitrate and ammonium during the second trimester.

**Table 2.** Postpartum Depression Associated With Air Pollution Exposure in Specified Periods of the Pregnancy

Pollutant^a^	Adjusted OR (95% CI)^b^					
	Entire pregnancy	First trimester	Second trimester	Third trimester	Third trimester + after delivery	During pregnancy + after delivery
Kriged air pollutants						
PM_2.5_	1.01 (0.99-1.03)	0.98 (0.96-1.00)	1.01 (0.99-1.03)	1.02 (1.00-1.04)^c^	1.02 (1.00-1.04)^c^	1.02 (1.00-1.03)^c^
PM_10_	1.02 (1.00-1.04)	0.99 (0.98-1.01)	1.01 (0.99-1.02)	1.02 (1.00-1.03)^c^	1.03 (1.01-1.04)^c^	1.02 (1.00-1.04)^c^
NO_2_	0.98 (0.95-1.00)	1.00 (0.98-1.02)	1.00 (0.98-1.03)	0.98 (0.96-1.00)	0.97 (0.95-1.00)	0.97 (0.94-1.00)
O_3_	1.07 (1.04-1.10)^c^	1.02 (1.00-1.04)^c^	1.02 (1.00-1.03)	1.04 (1.02-1.06)^c^	1.04 (1.02-1.05)^c^	1.09 (1.06-1.12)^c^
PM_2.5_ constituents						
PM_2.5_ total mass	1.02 (1.00-1.04)	0.99 (0.97-1.01)	1.02 (1.00-1.04)^c^	1.02 (0.99-1.04)	1.03 (1.00-1.05)^c^	1.03 (1.00-1.05)^c^
PM_2.5_ sulfate	1.00 (0.98-1.02)	0.99 (0.99-1.00)	1.00 (0.99-1.01)	1.01 (1.00-1.02)	1.02 (1.01-1.02)^c^	1.01 (1.00-1.03)
PM_2.5_ nitrate	1.01 (0.99-1.04)	0.99 (0.97-1.00)	1.02 (1.01-1.04)^c^	1.00 (0.99-1.02)	1.00 (0.99-1.02)	1.01 (0.99-1.04)
PM_2.5_ ammonium	1.02 (0.99-1.04)	0.99 (0.97-1.00)	1.02 (1.01-1.03)^c^	1.01 (0.99-1.02)	1.01 (0.99-1.03)	1.02 (0.99-1.04)
PM_2.5_ organic matter	1.02 (1.00-1.04)^c^	1.01 (0.99-1.02)	1.01 (0.99-1.03)	1.01 (0.99-1.02)	1.02 (1.00-1.03)	1.02 (1.00-1.05)^c^
PM_2.5_ black carbon	1.03 (1.00-1.06)^c^	1.03 (1.00-1.05)^c^	0.99 (0.97-1.03)	1.01 (0.99-1.05)	1.03 (1.00-1.06)^c^	1.04 (1.00-1.09)^c^

Abbreviations: NO_2_, nitrogen dioxide; O_3_, ozone; OR, odds ratio; PM_2.5_, particulate matter ≤2.5 μm; PM_10_, particulate matter ≤10 μm.

^a^ Units of measurement are units μg/m^3^ for PM_10_, PM_2.5_ total mass, and PM_2.5_ constituents, and parts per billion for NO_2_ and O_3_.

^b^ ORs and 95% CIs were calculated per IQR increment for each air pollutant. The base model was adjusted for maternal age, race and ethnicity, education, median neighborhood household income, smoking during pregnancy, season of conception, and year of infant birth; county was fitted as a random effect.

^c^ Significant at *P* < .05.

### Effect Modification by Maternal Characteristics

In subgroup analyses (eTable 4 in Supplement 1), significant heterogeneity in associations with different air pollutants among population subgroups was observed. Overall, the associations between antepartum and postpartum air pollution exposure and PPD were stronger among mothers aged 25 to 34 years, African American or Hispanic mothers, mothers with higher education (college or more), and mothers with underweight. While Cochran *Q* tests did not reveal any significant heterogeneity for other characteristics, we observed slightly higher associations among mothers who lived in low-income neighborhoods (quartiles 1 and 2).

## Discussion

To our knowledge, this study is the first to examine the association between antepartum and postpartum air pollution exposure, particularly PM_2.5_ chemical components, and PPD using a time-to-event framework. In this large obstetric population of 340 679 women in Southern California, we found that long-term antepartum and postpartum exposures to O_3_, PM_10_, and PM_2.5_ and its chemical constituents (organic matter and black carbon) were associated with an increased risk of PPD (from 2% for PM_2.5_ and PM_10_ to 9% for O_3_). Overall, an increased risk of PPD was associated with PM_10_ and PM_2.5_ exposure during the late pregnancy and postpartum periods and with O_3_ exposure during the entire pregnancy and postpartum period.

Although a few studies have investigated air pollution exposure and PPD, the findings are inconsistent due to limited relevant studies and large variations in PPD screening and ascertainment and exposure levels and windows among studies.^4,17,18,19^ Only 1 previous study assessed postpartum NO_2_ but reported no association with PPD.^18^ Most existing studies were conducted in regions with high air pollution concentrations, which were approximately 2 to 3 times that of our study.^4,17,19^ The only other study with air pollution levels similar to those in our study was conducted in urban Los Angeles.^18^ However, this study used the Center for Epidemiologic Studies-Depression Scale rather than PPD diagnosis and had a small sample size (n = 180). Our results are partially consistent with previous findings. We found that antepartum and postpartum exposures to PM_2.5_, PM_10_, and O_3_ were associated with an increased PPD risk, although the sensitive windows were different: O_3_ during the entire antepartum and the postpartum period and PM_10_ and PM_2.5_ during the late pregnancy and postpartum period. We did not observe an elevated risk of PPD to be associated with NO_2_ exposure. Compared with the limited studies, our study adds to the knowledge of associations between PPD and low levels of air pollution.

No prior research has explored the difference in concentrations and components of PM_2.5_. For trimester-specific exposures, we found that first-trimester PM_2.5_ black carbon and second-trimester PM_2.5_ nitrate and ammonium exposures were associated with an increased risk of PPD. For long-term exposures during pregnancy and postpartum periods, PM_2.5_ organic matter and black carbon were the main components associated with PPD.

Previous studies have proposed potential mechanisms that may support our findings, such as dysfunction of the HPA axis, neuroinflammation, and oxidative stress. For example, past research has shown that when exposed to PM_2.5_, the HPA axis becomes overactivated and cannot normally regulate the body’s stress response through negative feedback^12,13,18,37^ due to decreased glucocorticoid receptor levels and the subsequent release of extra cortisol.^37,38^ Particulate matter in the upper respiratory epithelium^39^ causes an inflammatory response that releases proinflammatory cytokines, such as interleukin 1α (IL-1α), IL-1β, IL-6, and tumor necrosis factor-α.^7,37,38,40^ The subsequent release of these proinflammatory cytokines can lead to increased activation of the HPA axis. As a reactive oxygen species, long-term O_3_ exposure can cause oxidative stress, leading to continued neuroinflammation,^10,41,42^ thus increasing proinflammatory cytokine levels.

Associations of demographic factors with PPD are mixed and complex.^6^ In our stratified analysis, the risk of PPD associated with air pollution exposure was significantly higher among mothers aged 25 to 34 years, African American or Hispanic mothers, mothers with higher education, and mothers with underweight, suggesting that these population subgroups may be more vulnerable to air pollution and at an increased risk for PPD. Effective screening and prevention strategies (eg, purposeful use of air filters, avoidance of outdoor activity during smoggy days, and avoidance of major sources of air pollution) focusing on the most influential time windows (ie, late pregnancy and after delivery) could be recommended, especially for vulnerable subpopulations, to optimize the benefits of reducing air pollution exposure on maternal mental health.

### Strengths and Limitations

The main strengths of this study include the large and diverse obstetric population; the high-quality and rich clinical data, especially PPD ascertainment; a wide range of air pollutants and exposure windows examined; detailed residential history in combination with well-validated air pollution models; and comprehensive individual- and neighborhood-level covariates that allowed us to control for a series of potential confounders, explore effect modification by maternal characteristics, and conduct several sensitivity analyses to check the robustness of our findings. Some limitations of our study also should be acknowledged. First, although residential mobility during pregnancy was considered, postpartum exposures were estimated solely based on maternal address at delivery, which may have led to exposure misclassification. Nonetheless, based on the residential history during pregnancy for this population, most women who relocated may have moved within the same subregion (median distance, 6 km), which would not significantly change their exposure levels.^32^ In addition, potential exposure misclassifications may also exist since indoor and personal exposure levels could not be estimated without data on personal monitors or activity patterns. Second, it is common for individuals with mental health disorders to have delays in diagnosis and initial treatment.^43^ Although we used follow-up data from multiple time points and detailed medical records, a proportion of the women with depression may not have been identified in a timely manner, which may have led to an underestimation of the associations. Future research may also consider PPD events over a longer postpartum period. Third, although several covariates were adjusted for, some residual or unmeasured covariates were inevitable due to data unavailability, such as psychiatric history, adverse life events, and marital status, which may affect mental health and potentially bias the estimates. Moreover, previous studies have reported associations between other environmental factors and PPD^44,45^; thus, research is needed to explore the joint effects of air pollution and other related exposures, such as green space, noise, and meteorologic factors. Furthermore, studies conducted in other regions and populations are warranted as air pollution levels and compositions could vary in different regions and be associated with different health outcomes.

## Conclusions

The findings of this large, retrospective cohort study provide insights into an association of long-term antepartum and postpartum air pollution exposure with PPD. Given that PPD prevalence is expected to increase over the next decade and may lead to heavier disease burden,^46^ identifying the modifiable environmental risk factors and developing interventions are important public health issues.

## Notes

Supplement 1. **eMethods.** Supplementary Methods **eResults.** Supplementary Results **eFigure.** Flow Diagram **eTable 1.** *ICD9/10* Diagnostic Codes and Medications Used to Ascertain Postpartum Depression Diagnosis **eTable 2.** Summary Statistics and Pearson Correlation Coefficients Between Air Pollutant Exposure Metrics During Pregnancy and the Postpartum Period **eTable 3.** Adjusted Odds Ratios (ORs) and 95% CIs of PPD Associated With Air Pollution Exposure **eTable 4.** Adjusted Odds Ratios (ORs) and 95% CIs of Postpartum Depression Associated With Postpartum Air Pollution Exposure Among Population Subgroups
eReferences

Supplement 2.
Data Sharing Statement
