# Supplementary material for: Association of Antepartum and Postpartum Air Pollution Exposure With Postpartum Depression in Southern California
Source: JAMA Netw Open. 2023 Oct 18;6(10):e2338315. doi: 10.1001/jamanetworkopen.2023.38315 (PMC10585409; doi:10.1001/jamanetworkopen.2023.38315)
Supplement: Supplement 1. — eMethods. Supplementary Methods eResults. Supplementary Results eFigure. Flow Diagram eTable 1. ICD9/10 Diagnostic Codes and Medications Used to Ascertain Postpartum Depression Diagnosis eTable 2. Summary Statistics and Pearson Correlation Coefficients Between Air Pollutant Exposure Metrics During Pregnancy and the Postpartum Period eTable 3. Adjusted Odds Ratios (ORs) and 95% CIs of PPD Associated With Air Pollution Exposure eTable 4. Adjusted Odds Ratios (ORs) and 95% CIs of Postpartum Depression Associated With Postpartum Air Pollution Exposure Among Population Subgroups eReferences [file jamanetwopen-e2338315-s001.pdf]

Supplemental Online Content

Sun Y, Headon KS, Jiao A, et al. Association of ante- and postpartum air pollution exposure with postpartum depression in Southern California. *JAMA Netw Open*. 2023;6(10):e2338315. doi:10.1001/jamanetworkopen.2023.38315

eMethods. Supplementary Methods
eResults. Supplementary Results
eFigure. Flow Diagram
eTable 1. *ICD9/10* Diagnostic Codes and Medications Used to Ascertain Postpartum Depression Diagnosis
eTable 2. Summary Statistics and Pearson Correlation Coefficients Between Air Pollutant Exposure Metrics During Pregnancy and the Postpartum Period
eTable 3. Adjusted Odds Ratios (ORs) and 95% CIs of PPD Associated With Air Pollution Exposure
eTable 4. Adjusted Odds Ratios (ORs) and 95% CIs of Postpartum Depression Associated With Postpartum Air Pollution Exposure Among Population Subgroups
eReferences

This supplemental material has been provided by the authors to give readers additional information about their work.

© 2023 Sun Y et al. *JAMA Network Open.*

eMethods. Supplementary Methods

Exposure assessment

The cross-validation R^2 of the empirical Bayesian kriging (EBK) model ranged from 0.65 to 0.75 for different air pollutants.[1] Further details of the EBK method have been previously described.[1,2] Publicly-available monthly $PM_{2.5}$ total mass and constituents, including sulfate, nitrate, ammonium, organic matter, and black carbon during the study period were derived from the fine-resolution geoscience-derived model. In our study region of the Southwestern United States, the $PM_{2.5}$ species model showed the highest cross-validated agreement for nitrate (R^2 = 0.78), followed by ammonium (R^2= 0.75), sulfate (R^2= 0.59), organic matter (R^2= 0.52), and black carbon (R^2= 0.42).[3,4] More details of this exposure measurement have been described elsewhere.[5]

Information on residential changes during pregnancy (address, start date, and end date) was abstracted from KPSC EHRs. As part of membership requirements, patient residential history was regularly updated with changes in residential address. This was available in structured format and used for this analysis. Monthly air pollution estimates during pregnancy were spatiotemporally linked to each woman based on the geocoded residential history; air pollution exposures during postpartum periods were estimated based on the residential address at delivery. The missing values of monthly concentrations were imputed using the average of the adjacent months. We then temporally interpolated the monthly air pollution metrics to generate daily values, and calculated trimester-specific and postpartum exposures by averaging the air pollution measurements in each specific time period: entire pregnancy (the date of conception to the date of delivery), the first trimester (1st - 3rd gestational months), second trimester (4th - 6th gestational months), third trimester (7th gestational months - the date of delivery), and the combination of the third trimester and postpartum periods. Long-term ante- and post-partum air pollution exposure was defined as the period from conception to the date of PPD diagnosis within 6-month postpartum. Gestational age was determined by the last menstrual period date and corroborated by early pregnancy ultrasonography.

Sensitivity analysis

In sensitivity analyses, we examined the influence of adjusting for pregnancy-related comorbidities (preeclampsia, gestational hypertension, and gestational diabetes) and preterm birth, or further excluding participants with dysthymia, adjustment disorder with depressed mood, and mild mental disorders diagnosed (n=1877, no overlap with other PPD diagnostic codes). We also performed sensitivity analyses adding green space exposure, which has been demonstrated as a potential risk factor for PPD in our prior study.[6] We used a validated machine learning model developed in our previous work to estimate green space exposure based on street view images within a 1 km radius around the residential address at delivery.[7] We further controlled for preconception air pollution exposure to account for air pollution levels one year before conception. We excluded infants conceived >19 weeks before 1 January 2008 (n=13,763) or <43 weeks before 31 December 2016 (n=3,030) to avoid fixed cohort bias as a sensitivity analysis. Women with multiple deliveries during the study period are represented in the study population; therefore, we added multiple deliveries as a random effect in the sensitivity analysis. Moreover, we conducted sensitivity analyses restricted to only urban zip codes due to reported urban-rural differences in air pollution levels and PPD frequency.[8] These differences may be explained by different factors influencing urban and rural populations (e.g., social support, issue of accessing healthcare).[8] Urban areas were defined as those with a rural-urban commuting area code of 1.0.[9]

© 2023 Sun Y et al. *JAMA Network Open.*

eResults. Supplementary Results

Summary statistics and Pearson correlation coefficients between air pollution metrics are shown in eTable 2. The median (IQR) concentration of maternal exposures to kriged $PM_{2.5}$, PM_{10}, NO_2, and O_3 during pregnancy and the postpartum period were 11.60 (3.06) µg/m^3, 27.63 (6.16) µg/m^3, 16.25 (5.38) ppb, and 42.88 (9.34) ppb, respectively. The concentration of $PM_{2.5}$ constituents was as follows (median [IQR], µg/m^3): $PM_{2.5}$ total mass, 13.14 (3.87); sulfate, 1.27 (0.27); nitrate, 2.45 (0.91); ammonium, 0.97 (0.38); organic matter, 5.46 (1.64); black carbon, 1.61 (1.01).

© 2023 Sun Y et al. *JAMA Network Open.*

eFigure. Flow Diagram

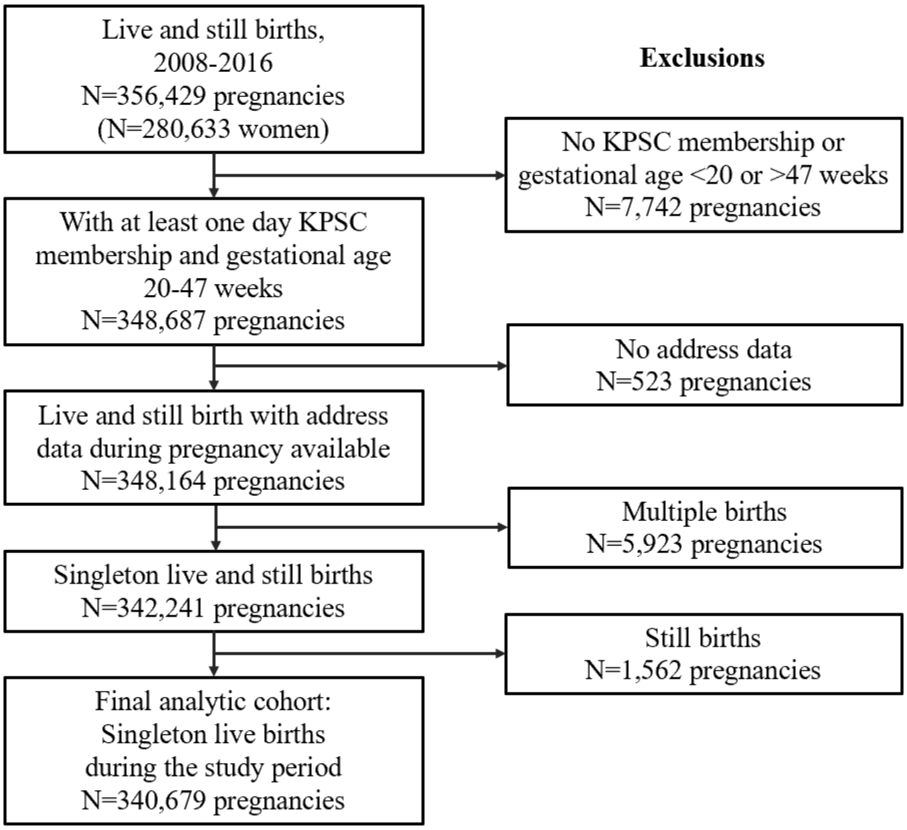

© 2023 Sun Y et al. *JAMA Network Open.*

eTable 1. ***ICD9/10*** **Diagnostic Codes and Medications Used to Ascertain Postpartum Depression Diagnosis**

ICD-9-CM Code	ICD-10-CM Code	Medications
300.4	F32.9	Bupropion
309.0	F33.0	Celexa
311	F33.2	Citalopram
	F33.3	Cymbalta
	F33.41	Desvenlafaxine
	F33.9	Duloxetine
	F34.1	Effexor
	F43.21	Escitalopram
	F53.0	Fluoxetine
		Lexapro
		Paroxetine
		Paxil
		Pristiq
		Prozac
		Sertraline
		Venlafaxine
		Wellbutrin
		Zoloft

© 2023 Sun Y et al. *JAMA Network Open.*

eTable 2. Summary Statistics and Pearson Correlation Coefficients Between Air Pollutant Exposure Metrics During Pregnancy and the Postpartum Period

						Kriged air pollutants [a]				$PM_{2.5}$ constituents [b]					
	Mean	Standard deviation	Median	IQR	N	Kriged $PM_{2.5}$	Kriged PM_{10}	Kriged NO_2	Kriged O_3	$PM_{2.5}$ total mass	$PM_{2.5}$ sulfate	$PM_{2.5}$ nitrate	$PM_{2.5}$ ammonium	$PM_{2.5}$ organic matter	$PM_{2.5}$ black carbon
**Kriged $PM_{2.5}$**	11.69	2.25	11.60	3.06	340619	1.00									
**Kriged PM_{10}**	28.53	5.17	27.63	6.16	340619	0.66	1.00								
**Kriged NO_2**	15.86	3.82	16.25	5.38	340619	0.64	0.30	1.00							
**Kriged O_3**	43.64	5.73	42.88	9.34	340619	-0.11	0.23	-0.33	1.00						
**$PM_{2.5}$ total mass**	12.95	2.53	13.14	3.87	340666	0.82	0.51	0.71	-0.05	1.00					
**$PM_{2.5}$ sulfate**	1.28	0.21	1.27	0.27	340666	0.58	0.54	0.42	0.15	0.58	1.00				
**$PM_{2.5}$ nitrate**	2.40	0.60	2.45	0.91	340666	0.79	0.56	0.60	-0.02	0.89	0.50	1.00			
**$PM_{2.5}$ ammonium**	0.97	0.30	0.97	0.38	340666	0.71	0.51	0.61	0.04	0.76	0.56	0.83	1.00		
**$PM_{2.5}$ organic matter**	5.44	1.24	5.46	1.64	340666	0.75	0.45	0.66	-0.06	0.93	0.47	0.72	0.57	1.00	
**$PM_{2.5}$ black carbon**	1.56	0.58	1.61	1.01	340666	0.62	0.16	0.67	-0.28	0.83	0.30	0.64	0.54	0.75	1.00

IQR, interquartile range. The units are $\mu g/m^3$ for PM_{10}, $PM_{2.5}$ and $PM_{2.5}$ constituents, and parts per billion for NO_2 and O_3.
a. air pollutants from kriging interpolation of Environmental Protection Agency's routine monitoring station data;
b. $PM_{2.5}$ constituents from a fine-resolution geoscience-derived model.

© 2023 Sun Y et al. *JAMA Network Open.*

eTable 3. Adjusted Odds Ratios (ORs) and 95% CIs of PPD Associated With Air Pollution Exposure

Air Pollution Exposure	OR	95% CI	
Main model + further adjusting for one-year preconception air pollution			
Kriged $PM_{2.5}$	1.04	1.01	1.07
Kriged PM_{10}	1.01	0.98	1.03
Kriged NO_2	1.00	0.96	1.05
Kriged O_3	1.05	1.01	1.09
$PM_{2.5}$ total mass	1.04	1.01	1.07
$PM_{2.5}$ sulfate	1.02	1.00	1.05
$PM_{2.5}$ nitrate	1.02	0.99	1.05
$PM_{2.5}$ ammonium	1.03	1.00	1.06
$PM_{2.5}$ organic matter	1.03	1.01	1.06
$PM_{2.5}$ black carbon	1.03	1.00	1.07
Main model + pregnancy-related comorbidities (preeclampsia, gestational hypertension and gestational diabetes) and preterm birth			
Kriged $PM_{2.5}$	1.02	1.00	1.04
Kriged PM_{10}	1.03	1.01	1.05
Kriged NO_2	0.97	0.94	1.00
Kriged O_3	1.08	1.04	1.11
$PM_{2.5}$ total mass	1.02	1.00	1.04
$PM_{2.5}$ sulfate	1.01	0.99	1.03
$PM_{2.5}$ nitrate	1.01	0.99	1.04
$PM_{2.5}$ ammonium	1.02	0.99	1.04
$PM_{2.5}$ organic matter	1.02	1.00	1.04
$PM_{2.5}$ black carbon	1.03	1.00	1.05
Main model + green space exposure (street total green space within 1 km)			
Kriged $PM_{2.5}$	1.02	1.00	1.03
Kriged PM_{10}	1.02	1.01	1.04
Kriged NO_2	0.97	0.94	1.00
Kriged O_3	1.09	1.06	1.13
$PM_{2.5}$ total mass	1.03	1.01	1.05
$PM_{2.5}$ sulfate	1.01	1.00	1.03
$PM_{2.5}$ nitrate	1.01	0.99	1.04
$PM_{2.5}$ ammonium	1.02	0.99	1.04
$PM_{2.5}$ organic matter	1.02	1.00	1.05
$PM_{2.5}$ black carbon	1.04	1.01	1.07
Main model + excluding infants conceived > 19 weeks before 1 January 2008 or < 43 weeks before 31 December 2016			
Kriged $PM_{2.5}$	1.02	1.00	1.05
Kriged PM_{10}	1.03	1.01	1.05
Kriged NO_2	0.98	0.95	1.00
Kriged O_3	1.09	1.05	1.12
$PM_{2.5}$ total mass	1.03	1.01	1.06
$PM_{2.5}$ sulfate	1.02	1.00	1.04
$PM_{2.5}$ nitrate	1.02	0.99	1.04
$PM_{2.5}$ ammonium	1.02	0.99	1.04
$PM_{2.5}$ organic matter	1.03	1.01	1.05
$PM_{2.5}$ black carbon	1.04	1.01	1.07

© 2023 Sun Y et al. *JAMA Network Open.*

Main model + excluding participants (n=1877, no overlap with other PPD diagnostic codes) with dysthymia, adjustment disorder with depressed mood, and mild mental disorders diagnosed			
Kriged $PM_{2.5}$	1.02	1.00	1.04
Kriged PM_{10}	1.02	1.00	1.04
Kriged NO_2	0.97	0.94	1.00
Kriged O_3	1.09	1.06	1.13
$PM_{2.5}$ total mass	1.03	1.00	1.05
$PM_{2.5}$ sulfate	1.01	0.99	1.03
$PM_{2.5}$ nitrate	1.02	0.99	1.04
$PM_{2.5}$ ammonium	1.02	0.99	1.04
$PM_{2.5}$ organic matter	1.02	1.00	1.05
$PM_{2.5}$ black carbon	1.04	1.01	1.05
Main model + multiple delivery as a random effect			
Kriged $PM_{2.5}$	1.02	1.00	1.03
Kriged PM_{10}	1.02	1.00	1.04
Kriged NO_2	0.97	0.94	1.00
Kriged O_3	1.09	1.06	1.12
$PM_{2.5}$ total mass	1.03	1.00	1.05
$PM_{2.5}$ sulfate	1.01	1.00	1.03
$PM_{2.5}$ nitrate	1.01	0.99	1.04
$PM_{2.5}$ ammonium	1.02	0.99	1.04
$PM_{2.5}$ organic matter	1.02	1.00	1.05
$PM_{2.5}$ black carbon	1.04	1.00	1.07
Main model, restricted to urban zip code			
Kriged $PM_{2.5}$	1.02	1.00	1.04
Kriged PM_{10}	1.03	1.01	1.05
Kriged NO_2	0.98	0.95	1.01
Kriged O_3	1.08	1.05	1.12
$PM_{2.5}$ total mass	1.04	1.01	1.06
$PM_{2.5}$ sulfate	1.02	1.00	1.04
$PM_{2.5}$ nitrate	1.02	1.00	1.05
$PM_{2.5}$ ammonium	1.03	1.01	1.06
$PM_{2.5}$ organic matter	1.03	1.01	1.05
$PM_{2.5}$ black carbon	1.05	1.01	1.08

PPD, postpartum depression; SD, standard deviation; ORs and 95% CIs were calculated for per interquartile range (IQR) increment for each air pollutant; Main model adjusted for maternal age, race/ethnicity, education, neighborhood household income, smoking during pregnancy, season of conception, and year of birth; county was fitted as a random effect.

The units are $\mu g/m^3$ for PM_{10}, $PM_{2.5}$ mass and $PM_{2.5}$ constituents, and parts per billion for NO_2 and O_3.

© 2023 Sun Y et al. *JAMA Network Open.*

eTable 4. Adjusted Odds Ratios (ORs) and 95% CIs of Postpartum Depression Associated With Postpartum Air Pollution Exposure Among Population Subgroups

Description [a]		ORs per IQR air pollutant metrics	95% CI		p value for Cochrane's Q test
**Maternal race/ethnicity**					
**Kriged $PM_{2.5}$**	African American	0.940	0.873	1.012	.14
	Asian	1.033	0.945	1.130	
	Hispanic	1.040	1.008	1.074	
	Non-Hispanic white	0.999	0.963	1.035	
	Multiple/other	1.009	0.897	1.131	
**Kriged PM_{10}**	African American	0.962	0.885	1.045	.20
	Asian	1.075	0.994	1.162	
	Hispanic	1.024	0.993	1.056	
	Non-Hispanic white	1.033	1.001	1.066	
	Multiple/other	1.097	0.994	1.210	
**Kriged NO_2**	African American	0.925	0.839	1.019	.54
	Asian	0.977	0.896	1.064	
	Hispanic	0.988	0.950	1.028	
	Non-Hispanic white	0.958	0.916	1.002	
	Multiple/other	0.954	0.848	1.074	
**Kriged O_3**	African American	1.218	1.115	1.331	.04
	Asian	1.044	0.942	1.158	
	Hispanic	1.064	1.020	1.111	
	Non-Hispanic white	1.078	1.020	1.139	
	Multiple/other	1.109	0.974	1.263	
**$PM_{2.5}$ total mass**	African American	0.959	0.874	1.052	<.001
	Asian	1.005	0.919	1.099	
	Hispanic	1.078	1.038	1.119	
	Non-Hispanic white	0.997	0.960	1.034	
	Multiple/other	0.996	0.882	1.125	
**$PM_{2.5}$ sulfate**	African American	1.060	0.988	1.139	.38
	Asian	1.033	0.962	1.109	
	Hispanic	1.025	0.997	1.054	
	Non-Hispanic white	1.005	0.975	1.035	
	Multiple/other	1.048	0.947	1.160	
**$PM_{2.5}$ nitrate**	African American	0.935	0.855	1.022	.02
	Asian	1.018	0.934	1.109	
	Hispanic	1.051	1.015	1.088	
	Non-Hispanic white	0.990	0.955	1.025	
	Multiple/other	1.040	0.926	1.167	
**$PM_{2.5}$ ammonium**	African American	1.000	0.916	1.091	.05
	Asian	1.022	0.948	1.103	
	Hispanic	1.051	1.016	1.087	
	Non-Hispanic white	0.981	0.947	1.017	
	Multiple/other	0.997	0.896	1.110	
**$PM_{2.5}$ organic matter**	African American	0.975	0.901	1.054	.01
	Asian	1.010	0.930	1.097	
	Hispanic	1.067	1.034	1.101	
	Non-Hispanic white	1.000	0.969	1.031	

© 2023 Sun Y et al. *JAMA Network Open.*

	Multiple/other	0.992	0.890	1.104	
**$PM_{2.5}$ black carbon**	African American	0.901	0.809	1.005	<.001
	Asian	0.953	0.861	1.055	
	Hispanic	1.084	1.035	1.134	
	Non-Hispanic white	1.011	0.961	1.065	
	Multiple/other	0.906	0.781	1.051	
**Maternal age**					
**Kriged $PM_{2.5}$**	< 25	0.979	0.927	1.031	.26
	25-34	1.023	0.995	1.052	
	≥ 35	1.015	0.966	1.065	
**Kriged PM_{10}**	< 25	0.974	0.928	1.022	.09
	25-34	1.030	1.003	1.057	
	≥ 35	1.040	0.998	1.085	
**Kriged NO_2**	< 25	0.908	0.850	0.970	.08
	25-34	0.989	0.956	1.024	
	≥ 35	0.973	0.923	1.025	
**Kriged O_3**	< 25	1.100	1.022	1.184	.34
	25-34	1.062	1.021	1.104	
	≥ 35	1.114	1.050	1.181	
**$PM_{2.5}$ total mass**	< 25	0.960	0.906	1.018	.02
	25-34	1.054	1.021	1.087	
	≥ 35	1.012	0.963	1.064	
**$PM_{2.5}$ sulfate**	< 25	0.972	0.929	1.017	.03
	25-34	1.034	1.005	1.065	
	≥ 35	1.040	1.001	1.081	
**$PM_{2.5}$ nitrate**	< 25	0.953	0.903	1.006	.08
	25-34	1.019	0.996	1.044	
	≥ 35	1.012	0.965	1.060	
**$PM_{2.5}$ ammonium**	< 25	0.958	0.907	1.012	.07
	25-34	1.028	1.000	1.058	
	≥ 35	1.029	0.984	1.077	
**$PM_{2.5}$ organic matter**	< 25	0.992	0.945	1.042	.03
	25-34	1.048	1.021	1.075	
	≥ 35	0.992	0.951	1.035	
**$PM_{2.5}$ black carbon**	< 25	0.938	0.869	1.012	<.001
	25-34	1.070	1.028	1.114	
	≥ 35	1.019	0.956	1.085	
**Pre-pregnancy body mass index (BMI) in categories**					
**Kriged $PM_{2.5}$**	Underweight	1.053	0.913	1.215	.83
	Normal	1.007	0.972	1.044	
	Overweight	1.024	0.983	1.066	
	Obese	1.006	0.969	1.044	
**Kriged PM_{10}**	Underweight	1.141	1.016	1.281	.24
	Normal	1.010	0.978	1.044	
	Overweight	1.017	0.979	1.057	
	Obese	1.029	0.993	1.067	
**Kriged NO_2**	Underweight	0.895	0.767	1.044	.77
	Normal	0.968	0.928	1.010	

© 2023 Sun Y et al. *JAMA Network Open.*

	Overweight	0.970	0.923	1.020	
	Obese	0.973	0.928	1.021	
**Kriged O_3**	Underweight	1.416	1.189	1.686	.01
	Normal	1.058	1.008	1.110	
	Overweight	1.075	1.017	1.136	
	Obese	1.107	1.051	1.167	
**$PM_{2.5}$ total mass**	Underweight	1.069	0.915	1.249	.53
	Normal	1.003	0.965	1.042	
	Overweight	1.038	0.992	1.087	
	Obese	1.039	0.995	1.085	
**$PM_{2.5}$ sulfate**	Underweight	1.087	0.956	1.236	.23
	Normal	0.994	0.965	1.023	
	Overweight	1.012	0.977	1.048	
	Obese	1.034	1.000	1.069	
**$PM_{2.5}$ nitrate**	Underweight	1.138	0.991	1.307	.29
	Normal	1.000	0.963	1.036	
	Overweight	1.027	0.984	1.072	
	Obese	1.007	0.968	1.048	
**$PM_{2.5}$ ammonium**	Underweight	1.199	1.053	1.364	.03
	Normal	1.019	0.984	1.056	
	Overweight	1.031	0.989	1.075	
	Obese	0.986	0.948	1.026	
**$PM_{2.5}$ organic matter**	Underweight	1.013	0.885	1.160	
	Normal	1.000	0.967	1.032	.29
	Overweight	1.032	0.993	1.072	
	Obese	1.047	1.010	1.086	
**$PM_{2.5}$ black carbon**	Underweight	0.969	0.805	1.166	.84
	Normal	1.025	0.974	1.078	
	Overweight	1.048	0.988	1.110	
	Obese	1.036	0.981	1.094	
**Household income**					
**Kriged $PM_{2.5}$**	Low (Q1-Q2)	1.021	0.990	1.053	.49
	High (Q3-Q4)	1.006	0.976	1.038	
**Kriged PM_{10}**	Low (Q1-Q2)	1.016	0.986	1.047	.41
	High (Q3-Q4)	1.033	1.004	1.062	
**Kriged NO_2**	Low (Q1-Q2)	0.973	0.933	1.014	.94
	High (Q3-Q4)	0.973	0.938	1.008	
**Kriged O_3**	Low (Q1-Q2)	1.109	1.061	1.160	.24
	High (Q3-Q4)	1.069	1.025	1.115	
**$PM_{2.5}$ total mass**	Low (Q1-Q2)	1.037	1.001	1.076	.57
	High (Q3-Q4)	1.023	0.990	1.057	
**$PM_{2.5}$ sulfate**	Low (Q1-Q2)	1.028	1.000	1.057	.32
	High (Q3-Q4)	1.011	0.986	1.036	
**$PM_{2.5}$ nitrate**	Low (Q1-Q2)	1.028	0.994	1.064	.37
	High (Q3-Q4)	1.008	0.978	1.039	
**$PM_{2.5}$ ammonium**	Low (Q1-Q2)	1.030	0.995	1.066	.40
	High (Q3-Q4)	1.016	0.986	1.046	
**$PM_{2.5}$ organic matter**	Low (Q1-Q2)	1.031	1.001	1.061	.64

© 2023 Sun Y et al. *JAMA Network Open.*

	High (Q3-Q4)	1.020	0.993	1.049	
**$PM_{2.5}$ black carbon**	Low (Q1-Q2)	1.043	0.995	1.092	.77
	High (Q3-Q4)	1.035	0.992	1.080	
**Maternal education**					
**Kriged $PM_{2.5}$**	< College	0.987	0.957	1.017	<.001
	≥ College	1.052	1.016	1.090	
**Kriged PM_{10}**	< College	1.003	0.977	1.030	.02
	≥ College	1.054	1.020	1.088	
**Kriged NO_2**	< College	0.962	0.926	0.998	.32
	≥ College	0.989	0.949	1.030	
**Kriged O_3**	< College	1.095	1.053	1.139	.57
	≥ College	1.076	1.026	1.127	
**$PM_{2.5}$ total mass**	< College	1.015	0.983	1.048	.15
	≥ College	1.052	1.013	1.092	
**$PM_{2.5}$ sulfate**	< College	0.991	0.967	1.015	<.001
	≥ College	1.049	1.020	1.079	
**$PM_{2.5}$ nitrate**	< College	1.007	0.978	1.037	.37
	≥ College	1.028	0.992	1.066	
**$PM_{2.5}$ ammonium**	< College	1.005	0.976	1.035	.18
	≥ College	1.036	1.002	1.072	
**$PM_{2.5}$ organic matter**	< College	1.018	0.991	1.045	.24
	≥ College	1.042	1.010	1.075	
**$PM_{2.5}$ black carbon**	< College	1.030	0.989	1.073	.45
	≥ College	1.054	1.005	1.106	

a. air pollutants from kriging interpolation of Environmental Protection Agency's routine monitoring station data, and $PM_{2.5}$ constituents from a fine-resolution geoscience-derived model.
HRs and 95% CIs were calculated for per interquartile range (IQR) increment for each air pollutant; Model adjusted for maternal age, race/ethnicity, education, neighborhood household income, smoking during pregnancy, season of conception, and year of birth; county was fitted as a random effect. Multiple/other: Pacific Islanders, Native American/Alaska Native and mothers with multiple race/ethnicities specified.

© 2023 Sun Y et al. *JAMA Network Open.*

eReferences

1. Wu J, Laurent O, Li L, et al., Adverse Reproductive Health Outcomes and Exposure to Gaseous and Particulate-Matter Air Pollution in Pregnant Women *Res Rep Health Eff Inst* 2016(188): p. 1-58.
2. Laurent O, Hu J, Li L, et al., Low birth weight and air pollution in California: Which sources and components drive the risk? *Environ Int* 2016b; 92-93: p. 471-7.DOI: 10.1016/j.envint.2016.04.034.
3. van Donkelaar A, Martin R V, Li C, et al., Regional Estimates of Chemical Composition of Fine Particulate Matter Using a Combined Geoscience-Statistical Method with Information from Satellites, Models, and Monitors *Environ Sci Technol* 2019; 53(5): p. 2595-2611.DOI: 10.1021/acs.est.8b06392.
4. Meng J, Li C, Martin R V, et al., Estimated Long-Term (1981-2016) Concentrations of Ambient Fine Particulate Matter across North America from Chemical Transport Modeling, Satellite Remote Sensing, and Ground-Based Measurements *Environ Sci Technol* 2019; 53(9): p. 5071-5079.DOI: 10.1021/acs.est.8b06875.
5. Sun Y, Li X, Benmarhnia T, et al., Exposure to air pollutant mixture and gestational diabetes mellitus in Southern California: Results from electronic health record data of a large pregnancy cohort *Environment International* 2021; 158: p. 106888.DOI: 10.1016/j.envint.2021.106888.
6. Sun Y, Molitor J, Benmarhnia T, et al., Association between urban green space and postpartum depression, and the role of physical activity: a retrospective cohort study in Southern California *The Lancet Regional Health - Americas* 2023: p. 100462.DOI: 10.1016/j.lana.2023.100462.
7. Sun Y, Wang X, Zhu J, et al., Using machine learning to examine street green space types at a high spatial resolution: Application in Los Angeles County on socioeconomic disparities in exposure *Science of The Total Environment* 2021; 787: p. 147653.DOI: 10.1016/j.scitotenv.2021.147653.
8. Vigod Sn Fau - Tarasoff L A, Tarasoff La Fau - Bryja B, Bryja B Fau - Dennis C-L, et al., Relation between place of residence and postpartum depression (1488-2329 (Electronic)).
9. U.S., DEPARTMENT OF AGRICULTURE. Rural-Urban Commuting Area (RUCA) Codes. 2010. https://www.ers.usda.gov/data-products/rural-urban-commuting-area-codes/ (accessed Jan 31, 2022).

© 2023 Sun Y et al. *JAMA Network Open.*
