# Supplementary material for: Association of Antepartum and Postpartum Air Pollution Exposure With Postpartum Depression in Southern California
Source: JAMA Netw Open. 2023 Oct 18;6(10):e2338315. doi: 10.1001/jamanetworkopen.2023.38315 (PMC10585409; doi:10.1001/jamanetworkopen.2023.38315)
Supplement: Supplement 2. — Data Sharing Statement [file jamanetwopen-e2338315-s002.pdf]

Data Sharing Statement

Sun. Association of Ante- and Postpartum Air Pollution Exposure With Postpartum Depression in Southern California. *JAMA Netw Open*. Published October 18, 2023. doi:10.1001/jamanetworkopen.2023.38315

Data
Data available: No

Additional Information
Explanation for why data not available: To protect confidentiality, the individual electronic medical record cannot be shared to the public.
